# Direct imaging of defect formation in strained organic flexible electronics by Scanning Kelvin Probe Microscopy

**DOI:** 10.1038/srep38203

**Published:** 2016-12-02

**Authors:** Tobias Cramer, Lorenzo Travaglini, Stefano Lai, Luca Patruno, Stefano de Miranda, Annalisa Bonfiglio, Piero Cosseddu, Beatrice Fraboni

**Affiliations:** 1 Department of Physics and Astronomy, University of Bologna, Viale Berti Pichat 6/2, Italy; 2 Department of Electric and Electronic Engineering, University of Cagliari, Piazza d’Armi, Italy; 3 DICAM, University of Bologna, Viale Risorgimento 2, Italy

## Abstract

The development of new materials and devices for flexible electronics depends crucially on the understanding of how strain affects electronic material properties at the nano-scale. Scanning Kelvin-Probe Microscopy (SKPM) is a unique technique for nanoelectronic investigations as it combines non-invasive measurement of surface topography and surface electrical potential. Here we show that SKPM in non-contact mode is feasible on deformed flexible samples and allows to identify strain induced electronic defects. As an example we apply the technique to investigate the strain response of organic thin film transistors containing TIPS-pentacene patterned on polymer foils. Controlled surface strain is induced in the semiconducting layer by bending the transistor substrate. The amount of local strain is quantified by a mathematical model describing the bending mechanics. We find that the step-wise reduction of device performance at critical bending radii is caused by the formation of nano-cracks in the microcrystal morphology of the TIPS-pentacene film. The cracks are easily identified due to the abrupt variation in SKPM surface potential caused by a local increase in resistance. Importantly, the strong surface adhesion of microcrystals to the elastic dielectric allows to maintain a conductive path also after fracture thus providing the opportunity to attenuate strain effects.

The objective of flexible electronics research is to establish a new technology capable to fabricate rugged electronic devices that operate steadily under mechanical deformation and that can be patterned on various thin and deformable substrates. A wide range of different material platforms including carbon-based nanostructures^1^,^2^,^3^ or organic compounds^4^,^5^,^6^, metals^7^,^8^ and oxides^9^ are currently investigated to realize devices that would be key to several emerging applications often related to the interface between humans and communication and information technology. Examples are wearable electronic transducers and processors or electronic energy harvesting systems as employed in electronic skin^10^,^11^,^12^,^13^,^14^, implantable bioelectronic devices^15^,^16^,^17^ or electronic textiles^7^. Progress in flexible electronics relies on the development of electronic materials and devices resistant to mechanical deformation. In regard of this objective, it is fundamental to study electronic material properties under strain at the micro- and nanoscale. Optical and electronic microscopies have been employed to investigate morphologic changes in the active layer of devices while exposed to strain^8^,^9^,^10^,^18^,^19^,^20^,^21^,^22^. This information was then correlated to electronic device properties measured at the same time and this approach allowed for example to investigate the relevance of plastic deformation, crack-formation and fragmentation in flexible and stretchable electronics^9^,^23^,^24^,^25^,^26^. Recently, atomic force microscopy was exploited to investigate strain effects in active layers. Nanometer scale changes in morphology at grain-boundaries^27^ or out-of-plain movements of nano-mesh electrodes^28^ were visualized as a consequence of strain. Here, we extend the methodologies of investigating strain effects in flexible electronics by applying Scanning Kelvin Probe Microscopy (SKPM) directly on mechanically strained organic thin film transistors (OTFT). The possibility to measure locally the impact of strain on electronic properties and morphology allows us to elucidate the microscopic mechanisms deciding on stress stability or failure in semiconducting thin films.

SKPM provides nanoscale mapping of the local electrostatic surface potential *V*_*SP*_(x), a key microscopic quantity characterizing charge carrier accumulation and transport in thin film transistors^29^,^30^,^31^. The gradient of the surface potential is equal to the local electric field in the channel that drives carrier transport^32^,^33^. SKPM on TFT structures allows further to measure the local charge density and can be exploited to provide detailed information on the shape of the bandtails in the semiconductors density of states^34^. Combined knowledge of the electric field and current density provides insight into the local sheet resistance and carrier mobility^35^. For example local increases in electric field result from transport barriers such as grain boundaries^36^ or electrode contacts^30^ and can therefore directly be revealed and quantified by SKPM^32^. These capabilities make SKPM a unique tool to investigate changes in local electronic properties caused by strain as has been shown recently by characterizing the impact of thermal expansion induced strain on the workfunction of Rubrene single crystals^37^.

Organic electronics provides an established material platform for flexible electronics devices that is compatible to low-cost, large area deposition by printing techniques. Solution processed semiconducting thin films containing either small molecule^38^ or polymeric semiconductors^4^ have been exploited to realize OTFTs and sensors^39^ with constant performance under mechanical deformation^40^. Several investigations have been undertaken to elucidate the mechanical properties of and defect formation in organic semiconducting thin films and provide crucial insight towards the realization of materials combining excellent charge transport properties with strain resistance^5^,^18^,^27^. Micro-crystalline semiconductors such as Bis (triisopropylsilylethnyl) pentacene (TIPS pentacene) are of particular interest as they integrate facile processing and high carrier mobility^41^. However, although stable flexible electronic circuits have been realized based on these materials, it has also been shown that micro crystalline thin films are affected by morphological changes and crack formation when subjected to strain caused for example by bending or thermal expansion^18^,^27^,^42^. Details on how local defects caused by strain impact on charge transport and whether they limit the application of micro-crystalline thin films in flexible electronics are still open issues.

In this manuscript we adopt the SKPM technique to be applied on deformed organic thin film transistors employing microcrystalline TIPS pentacene layer as semiconductor. The combination of macroscopic transport measurements with high resolution mapping of morphology and surface potential allows us to investigate the details on strain induced nano-crack formation and its impact on charge transport properties. Our results demonstrate that SKPM is feasible on flexible, deformed substrates thus becoming a unique tool to investigate local defect formation in flexible electronics.

## Results

### Flexible low-voltage organic OTFTs and force microscopy setup

**Figure 1:**
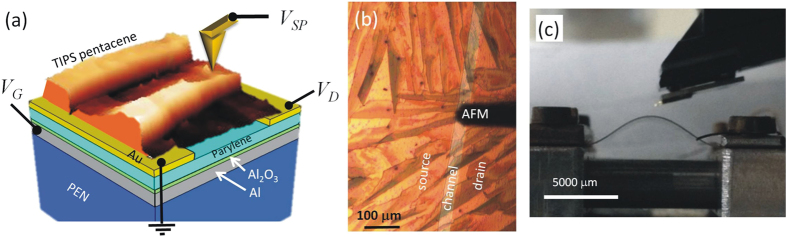
OTFT structure and setup for force microscopy on bent substrates. (**a**) scheme showing the transistor structure, applied voltages in SKPM mode and a 3D rendered AFM topography of two semiconducting crystals bridging the transistor channel; (**b**) optical micrograph of micro-crystalline TIPS-pentacene thin film deposited on transistor channel and inspected by force microscopy. The AFM cantilever is visible in the image; (**c**) photography of sample holder with bent transistor (*r*_*c*_ = 5.6 mm) below the force microscope’s probe hand.

The layer structure of the investigated OTFTs and the experimental setup for force microscopy on bent transistors is illustrated in Fig. 1. The investigated OTFTs were fabricated on 125 μm Polyethylenenaphthalate (PEN) as substrate and contain 90 nm Aluminum as gate electrode, 6 nm of Aluminum oxide and 170 nm of Parylene C as dielectric, 80 nm of gold as source and drain electrodes and a final layer of TIPS-pentacene as semiconductor. The latter was deposited by drop casting from a solution of 1 wt% in Anisole yielding a microcrystalline thin film. The electrical connections to operate the OTFT on the AFM stage are reported in Fig. 1a where *V*_*D*_, *V*_*G*_ and *V*_*SP*_(x) denote drain, gate and surface potential as determined in SKPM-mode, respectively. The 3D rendered AFM topography (Fig. 1a) and optical microscopy (Fig. 1b) reveal the typical dimensions of TIPS-pentacene micro-crystals to be 100 to 300 nm in height, 10–50 μm in width and up to several 100 μm in length. The OTFT channel contains micro-crystals bridging source and drain contacts and areas of uncovered dielectric giving rise to an overall hole carrier mobility of 0.05 cm^2^/Vs in these devices. In order to induce uniaxial tensile surface strain in the microcrystalline film, OTFT substrates were fixed to the two clamps of a dedicated sample holder. Reduction of the distance *l* between clamps induced bending of the transistor substrate as shown in Fig. 1c. Stable AFM acquisitions on these bent structures were possible when tip sample interaction was kept low by operating in non-contact mode and when the investigated region was situated on the topmost part of the bent transistor.

### Surface strain during bending of transistors

**Figure 2:**
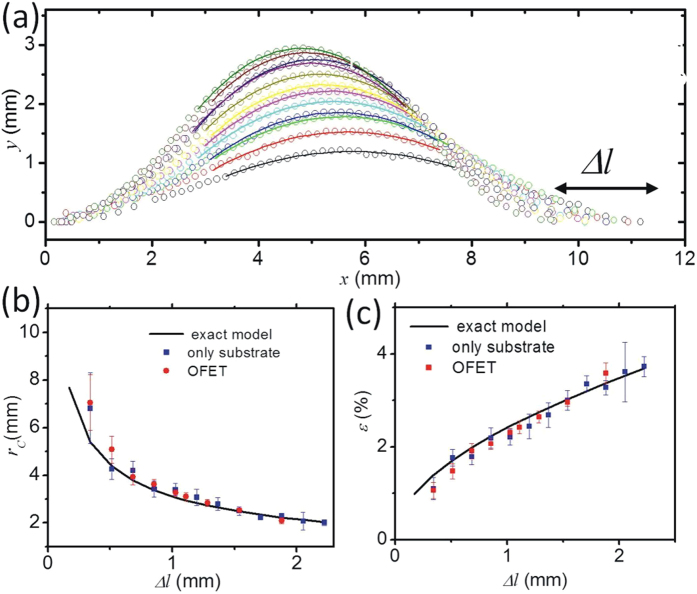
Mechanical deformation of flexible OTFT during AFM experiments. (**a**) traced profiles and fit to polynomial to extract the bending radius; (**b**) bending radius and (**c**) surface strain as a function of the lateral displacements of the clamps fixing the ends of the flexible OFET substrate. Data is compared to PEN substrate only and to a mathematical model describing substrate deflection.

To characterize the tensile strain exerted on the exposed microcrystalline thin film, we traced optically the profiles *y*(*x*) of the bent device as shown in Fig. 2a. OTFT electrodes and channel extend in these profiles over a length of 1.4 mm centered at the maximum of the deformed substrate. For the small area covered by the transistor, we extract a single radius of curvature *r*_*C*_ = −(*d*^2^*y/dx*^2^)^−1^ at the maximum of the profile. Values for *r*_*C*_ are plotted in Fig. 2b as a function of the distance variation between the two clamps, *Δl*. Assuming that the additional layers of the transistor are of negligible impact for the deformation (here t_device_/*t*_*S *_< 0.004), strain *ε* in the outermost microcrystalline layer is calculated by *ε *= *t*_*S*_/2*r*_*C*_ with *t*_*S*_ being the layer thickness of the substrate (125 μm)^20^,^43^, see Fig. 2c. For comparison, in Fig. 2b,c, we show also the deformation and surface strain of a PEN substrate without transistor as well as the exact mathematical solution of the differential equations describing the deflection of an ideal homogeneous, elastic substrate (see Supp. Inf. equation S12). For both cases no significant deviation in *r*_*C*_ or *ε* is observed with respect to the device deformation. After bending the substrate foils returned to a strain free flat geometry and we exclude viscoelastic deformations or polymer fatique from our considerations.

### Electrical performance of strained transistors

**Figure 3:**
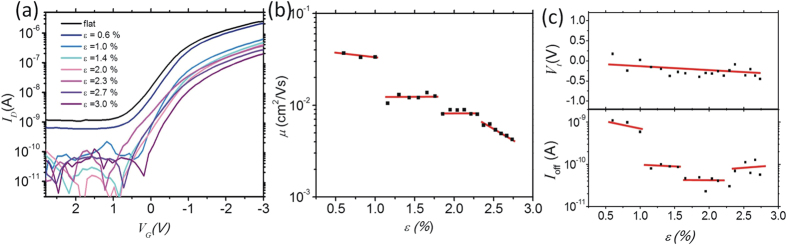
Electrical characteristics of strained OTFT. (**a**) Transfer characteristics at different strain values; (**b**,**c**) mobility μ, threshold Vt and off-current values Ioff as a function of strain. Intervals where electrical performance is less affected by strain are marked by red lines as guide to the eye.

The macroscopic electrical transistor properties were monitored during increasing mechanical deformation. Transistor transfer curves measured in saturation regime (*V*_*D*_ = −5 V) at characteristic strain values are reported in Fig. 3a. In all curves, carrier accumulation at negative gate potentials due to field effect is observed although a strong reduction in drain current with increasing strain is present. From the transfer-curves we calculated the mobility *μ*, the threshold voltage *V*_*t*_ and the off-current *I*_*off*_ which are depicted in Fig. 3b and c as a function of strain. Several important observations are made from these plots: *μ* and *I*_*off*_ show large discontinuous changes at specific strain values which permit to define four intervals as indicated in the figure. Within the intervals, transport parameters vary only gradually or even remain constant, whereas close to the interval boundaries large variations are observed. The initial discontinuity in *I*_*off*_ reaches an order of magnitude and exceeds its counterparts in *μ*. At higher strain values, *μ* continues to decrease whereas *I*_*off*_ remains almost constant and even shows a small increase at *ε* > 2.3%. In contrast, the threshold voltage *V*_*t*_ is not affected by strain and exhibits only a slight drift towards negative values over the full strain range. These observations clearly point to the formation of transport barriers in the semiconducting film that cause the reduction of *μ*. The barriers do not introduce additional trap states in the density of states of the semiconductor as *V*_*th*_ remains constant. In the following we investigate the microscopic origins of these barriers. The combined findings of micro- and macroscopic investigations and their detailed implications on transport are then discussed in the Discussion section below.

### Microscopic analysis of strained transistors

Parallel to the electrical transistor characteristics we acquired topography and surface potential maps on microcrystals by SKPM at increasing strain values. In SKPM a small AC voltage is applied to the conducting tip to modulate exclusively the electrostatic tip-sample interaction. This allows to use a lockin amplifier to separate the electrostatic forces from the overall forces acting between tip and sample. The surface potential *V*_*SP*_ is then obtained by a feedback loop programmed to nullify the electrostatic interaction by imposing a DC voltage offset between tip and sample. Variations in this offset are equal to variations in *V*_*SP*_ of the sample and are recorded during the image scan. In this way maps of *V*_*SP*_ as well as of the sample topography are created simultaneously in the SKPM scan. For thin film transistors exposing a semiconducting film, *V*_*SP*_ is defined when the semiconductor is rendered conductive, hence when field effect is used to accumulate charge carriers. In this case *V*_*SP*_ follows the electrostatic potential in the accumulation layer plus an additional offset due to differences in workfunction^29^,^44^.

**Figure 4:**
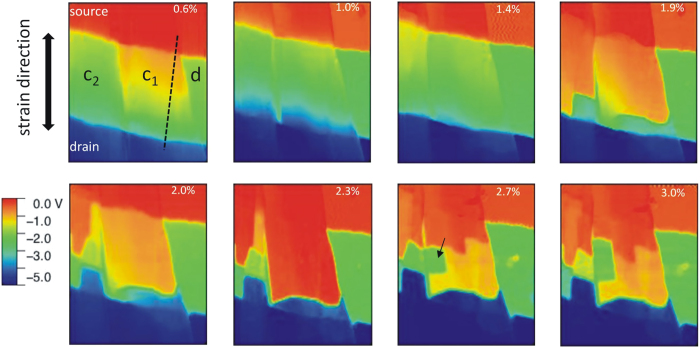
Maps of surface potential from Scanning Kelvin probe microscopy performed on a biased OTFT (VS = 0 V, VD = 5 V, VG = −3 V) at increasing strain *ε*. Symbols in the first map indicate areas covered by crystals (c1, c2) or with exposed dielectric (**d**).

The flexible substrate structure (Fig. 1c) requires low interaction forces to avoid deformations during AFM scans. This becomes feasible when operating the AFM in non-contact mode and we obtained stable image acquisition for different bending radii, provided that the mapped region was exposed at the highest point of the bent device. During the SKPM scan, the transistor was kept at constant drain and gate bias of *V*_*D*_ = −5 V and *V*_*G*_ = −3 V to drive a saturation current through the strained semiconducting microcrystals. In saturation regime *V*_*SP*_ follows the electrostatic potential in the accumulation layer in the semiconductor, thus allowing to draw conclusions on local carrier concentration and variations in resistance^29^,^31^,^44^. Figure 4a shows the resulting maps of *V*_*SP*_ at different characteristic strain values. Source and drain electrodes can easily be distinguished in these plots as they appear as equipotential surfaces at the values imposed by the source measure unit (*V*_*S*_ = 0 V, *V*_*D*_ = −5 V) plus an offset due to contact potential differences to the AFM tip. In the channel region the situation is more complex. Comparing to the topographic AFM image of the very same region as shown in Fig. 1a, it is evident that part of the channel region is not covered by organic semiconductor. In these areas (indicated by the letter d in the first SKPM map) the local surface potential is constant and not affected by strain. Its value results from interaction between the tip and the underlying gate electrode thus establishing a constant value of −2.2 V which is the sum of the imposed gate potential *V*_*G*_ = −3 V and an offset of around 0.8 V due to the workfunction difference between AFM tip (gold) and gate (Aluminum). Areas covered by organic crystals (marked with c_1_ and c_2_) instead show a different behavior and manifold changes are observed during increase of strain reflecting the formation of local barriers for charge transport. Initially changes in *V*_*SP*_ take place at the contacts between microcrystals and source and drain electrodes. At higher strain (*ε* = 1.9%) strong discontinuities and steps in *V*_*SP*_ appear on microcrystals in the channel. The majority of discontinuities follows initially a direction orthogonal to the strain direction (already existing discontinuities in *V*_*SP*_ are due to the borders of microcrystals). However, at larger strain values, discontinuities start to appear also in directions parallel to the strain (c_2_ ε = 2.0%; c_1_ ε = 2.7%). This leads to the formation of independent patches on the surface of microcrystals with constant *V*_*SP*_. Some of these patches in the SKPM image seem to be electrically isolated as they exhibit a *V*_*SP*_ value identical to areas not covered by semiconductor (marked by a black arrow in the map at ε = 2.7%).

**Figure 5:**
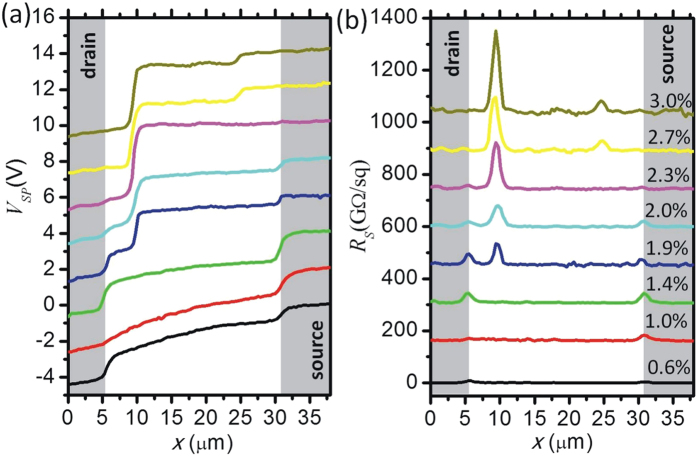
Analysis of Scanning Kelvin Probe microscopy data obtained on OTFT channel exposed to increasing strain. (**a**) profiles of surface potential VSP(x) across the transistor channel; (**b**) profiles of local sheet resistance Rs. Curves taken at higher strain are offset by ΔVSP = 2 V and ΔRS = 150 G Ω. Gate and drain potential were kept at VG = −3 V and VD = −5 V.

To achieve a quantitative interpretation of the effects we analyze the *V*_*SP*_ profiles along the shortest path crossing the channel as shown in Fig. 5a. The current density *J* along this path is assumed to be constant and to scale with the width *W* of the transistor channel. Consequently the local sheet resistance *R*_*S*_*(x)* can be calculated according to


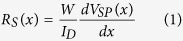


The *R*_*S*_ profiles shown in Fig. 5b allow to compare the impact of different defects. At the lowest strain value (*ε *= 0.6%) the *V*_*SP*_ profile still follows the superlinear shape typical for saturation regime with maximum field strength close to the drain electrode where the depletion in carrier density gives rise to small increase in *R*_*S*_. In addition a local increase in *R*_*S*_ due to contact resistance is present at the source electrode. The *R*_*S*_ profiles at *ε *= 1.0% and 1.4% show that initial strain effects take place at the source and drain electrode contacts leading to an increase in the resistance associated to charge injection and extraction. Further increasing strain (*ε *= 1.9% then gives rise to the formation of a defect in the channel with high local *R*_*S*_. Interestingly, the increasing strain does not further weaken the contacts. The defect in the channel dominates the overall resistance until at higher strain (*ε *= 2.7% a second defect with large local increase in *R*_*S*_ is formed.

**Figure 6:**
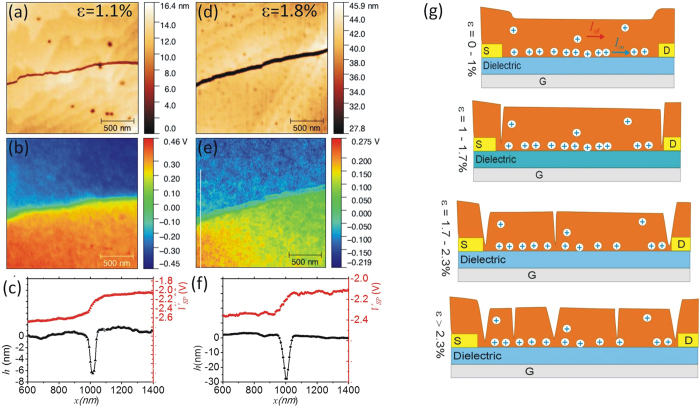
Crack formation in strained microcrystal as observed in AFM experiments on biased transistors and model for OTFT strain response. (**a**,**d**) AFM surface topography; (**b**,**e**) SKPM surface potential and (**c**,**f**) profiles of height and surface potential across the crack at the two different investigated strain values; (**g**) Model explaining the OTFT response to tensile strain.

The origin of *V*_*SP*_ discontinuities was then investigated by performing high resolution scans in SKPM mode. Typical maps obtained on the same area at different strain values (Fig. 6a–f) demonstrate that transport barriers arise due to crack formation in microcrystals. The overlapped profiles of surface height and surface potential (Fig. 6c) show a perfect correlation between the crack position and the drop in surface potential. Observing the same crack at a higher strain (Fig. 6d–f) demonstrates further that the mechanical deformation due to the strain is absorbed in an increased opening of the crack. Note that the drop in surface potential at higher strain is reduced due to the overall lower current density.

## Discussion

The presented microscopic surface potential maps combined with macroscopic electrical properties give a unique opportunity to analyze strain effects in flexible thin film transistors. For the investigated microcrystalline thin-film, we find a discontinuous, complex degradation of transistor performance when a pristine OTFT is subjected to increasing surface strain. At the same time, at the microscale we observe the formation of nano-cracks that locally increase sheet resistance over orders of magnitude. Nano-crack formation coincides with sharp drops in transistor transport properties, revealing it as the fundamental cause for OTFT degradation. However, our SKPM data also shows that nano-cracks do not interrupt transport completely but act as a local barriers thus giving rise to a stepwise more complex degradation.

Combining the different observations made at the micro- and macro-scale we develop the following model to explain the transistors response to tensile strain (Fig. 6g): At low strain values (*ε*  < 1%) the microcrystals adapt with elastic deformation to the surface strain and no defect formation is observed. Mobility varies gradually as strain leads to an increase in intermolecular distances and hence reduces electronic interactions. Such a finding has been described for different crystalline organic semiconductors such as Rubrene^45^. In this regime the devices also resist to extensive bending or deformation cycles^27^,^46^. At a critical strain value of *ε* ~1% irreversible changes occur and a first strong drop in transistor current is observed. We associate this drop in transistor performance to crack formation. A nano-meter sized crack clearly represents a barrier for transport that reduces mobility. However it does not introduce additional trap states and the density of states of the semiconductor remains unaltered, in agreement with the findings from transistor characterizations. The first cracks appear at the source and drain electrode contacts thus giving rise to an increased contact resistance. Initial defect formation is more likely to occur close to the electrode contact due to the presence of a step in the underlying surface topography caused by the presence of the bottom electrode. Microcrystals grown on this step get pre-strained as they adapt to the surface topography thus reducing the threshold for crack formation. After the first crack formation at *ε* ~1%, the transistor performance remains relatively stable as existing cracks absorb the deformation until at *ε* = 1.9% when new crack formation sets in and causes a second disruptive change in transport properties. Again the new cracks are able to absorb the deformation and to stabilize the strain response until at *ε * > 2.3 more complex fractures of the microcrystal start to appear. The complexity and the directionality of the multitude of crystal fractures which appear in this regime is determined by details of the shape, thickness and pre-existing defects of each individual microcrystal. In particular, due to variations in thickness of single microcrystals, cracks also appear in the direction parallel to the applied strain. At this stage cracks interrupt electrical transport severely and independent patches with constant surface potential emerge. These isolated patches are associated to the observed increase in off-current at high strain values: Due to the very weak electrical connection, charging and de-charging processes of these patches are very slow and during the fast transfer-sweep they act as charge carrier reservoirs contributing to a measurable drain current also in the off-state of the transistor.

Our findings allow us to draw some further conclusions about the cracks that penetrate the microcrystals. Crack openings at the exposed interface as observed by AFM are several tens of nanometers wide, clearly blocking transport completely. However, we hypothesize, that the adhesion of the microcrystals to the dielectric reduces or even prevents crack opening to occur at the buried interface with the gate dielectric as indicated in the Fig. 6g. The reason could be that the adhesion to the elastic surface limits the localization of strain responsible for crack formation and propagation^19^,^25^,^26^. The resulting asymmetric crack structure could explain why transport in the very confined accumulation layer close to the gate dielectric gets reduced, but not completely blocked during crack formation. Instead, the impact of cracks on the OTFT off-current is stronger, as the related doping and transport occur in the bulk of the crystal. Further it gets clear that crack formation only impacts on transport properties of micro-crystals, but does not lead to the formation of traps. In accordance to this, *V*_*th*_ remains constant as a function of strain.

The proposed model about strain response has several important implications on flexible organic electronics that employ microcrystalline organic thin films. The investigated TIPS pentacene is a compound with model character for organic semiconductors. Similar to other organic semiconducting crystals, intermolecular binding relies on weak van-der-Waals interactions. The interdigitated binding structure of the bulky sidechains in the crystal structure provides interactions in all spatial directions with comparable surface energies for different crystal planes^42^. These interactions combined with the long-range order of the crystal render the material susceptible to crack formation thus limiting the strain-interval in which reversible elastic response to tensile strain occurs^47^. Beyond a critical value of the range of *ε* ~1% irreversible changes occur due to the onset of crack formation and transport in crystals gets reduced. Therefore, in order to achieve best possible transport properties in organic flexible electronics, two well-known options are available: either ultra-thin substrates have to be employed that reduce surface strain during bending or sandwich architectures have to be used that position the sensitive micro-crystalline layer in the shear neutral plane^6^. However, if some degradation in transport properties can be accepted, then nano-crack formation is not in conflict with flexible electronics applications: once the cracks are formed in an initial strain cycle, the device response stabilizes as long as the adhesion of microcrystals to the underlying gate dielectric is strong enough. Such an approach of pre-straining induced crack formation has already been applied to improve the strain response of organic transistors^4^ or sensors^8^.

## Conclusions

In this work we introduce Scanning Kelvin Probe Microscopy (SKPM) on mechanically deformed flexible electronics devices as a new tool to characterize strain induced defect formation at the nanoscale. We employ the method to study the strain response of flexible organic thin film transistors (OTFT) containing a microcrystalline TIPS-pentacene thin film as semiconducting layer. Our results demonstrate that beyond a critical tensile strain value of *ε* ~1%, irreversible device degradation sets in due to nano-crack formation. SKPM allows to quantify the local increase in electric field-stength that occurs in nano-cracks and to map the related local increase in sheet resistance. The unique possibility to obtain a consecutive sequence of SKPM surface potential maps on the same micrometric area at increasing surface strain allows to unequivocally associate the formation of barriers to strain effects and to exclude possible effects due to material impurities. Based on our findings we propose a model for nano-crack generation in van-der-Waals microcrystals adherent to a gate dielectric that explains the strain related changes in transistor properties. We propose that degradation is not a continuous but instead a disruptive process: initially only at critical strain values, cracks are formed and transport decreases; in-between the critical values existing cracks absorb the mechanical deformation giving rise to a more stable response. We explain the fact that nano-cracks sustain a reduced transport although crack openings at the exposed surface show several tens of nanometer width by an asymmetric crack structure in which adhesion forces at the buried interface with the elastic gate dielectric prevent the localization of strain and crack opening. Therefore, the combined investigation of strain effects by macroscopic electrical characterization and microscopic mapping techniques allows an in-depth understanding of the related defect formation, necessary to improve materials and device architectures for future flexible electronics applications.

## Methods

### Tips-Pentacene OTFTs

125 μm-thick polyethylene naphtalate (PEN) films were cleaned by rinsing with acetone, isopropyl alcohol and deionized water. A 90 nm-thick layer of Al film was was deposited by thermal evaporation. Positive photoresist (Microposit AZ1518, 1500 rpm for 1 min; dried at 40 °C for 30 minutes) was exposed to UV through an acetate shadow mask. After development in NaOH (175 mM), the excess of aluminum was removed by 1% HF solution. Exposure to UV-produced ozone (1 hour in ambient condition) then resulted in a 6 nm-thick oxide layer on the Al gate electrode. Subsequently 170 nm-thick Parylene C layer were deposited by CVD. Source and drain electrodes were patterned into an interdigitated geometry (W = 23120 μm, L = 25 μm – see Supp. Inf.) by the same photolithographic process described above from an unique, 80 nm-thick gold layer deposited by thermal evaporation using aqueous KI solution for etching. TIPS pentacene (1 wt% in anisole) was deposited by drop casting at room temperature.

**Electrical characterization and SKPM** was performed with a Keysight B2912A source measure unit under ambient conditions. A Park NX10 Atomic force microscope equipped with PPP-NCSTAu probes (Nanosensors) was used for scanning probe characterizations. The sample holder (see Supp. Inf.) allowing bending and electrical connection of OTFTs was mounted onto the xy-scanner of the instrument. SKPM was operated in non-contact regime using a free oscilation amplitude *A*_*0*_ of 25 nm and a setpoint *A* = 0.9 *A*_*0*_. To stabilize performance in the attractive regime, the cantilever is driven at a frequency slightly larger than the fundamental resonant frequency. In non-contact mode the wear of the tip is minimized and a single cantilever is used for the full characterization of a sample at various strain values. To probe electrostatic forces, an AC bias of 1 V amplitude at a frequency of 17 kHz was applied to the tip. The the AC-bias driving frequency is distant from any resonance phenomena of the tip-cantilever system to reduce the impact of non-local electrostatic interactions on SKPM response. The resulting tip oscillation is fed after lock-in amplification into a feedback loop which is engaged to null the electrostatic interaction by adjusting a DC voltage offset applied to the tip. The resulting DC-voltage, called surface potential *V*_*SP*_ in the following, is recorded with the image topography. All *V*_*SP*_ data is shown as recorded. Topography images were corrected for a tilted plane background.

## Additional Information

**How to cite this article**: Cramer, T. *et al*. Direct imaging of Defect Formation in Strained Organic Flexible Electronics by Scanning Kelvin Probe Microscopy. *Sci. Rep.* **6**, 38203; doi: 10.1038/srep38203 (2016).

**Publisher's note:** Springer Nature remains neutral with regard to jurisdictional claims in published maps and institutional affiliations.

## Supplementary Material

Supplementary Information
