# Supplementary material for: Direct imaging of defect formation in strained organic flexible electronics by Scanning Kelvin Probe Microscopy
Source: Sci Rep. 2016 Dec 2;6:38203. doi: 10.1038/srep38203 (PMC5133578; doi:10.1038/srep38203)
Supplement: Supplementary Information [file srep38203-s1.pdf]

Supplementary Information

Direct Imaging of defect formation in strained organic flexible electronics by Scanning Kelvin Probe Microscopy

Tobias Cramer[*,a], Lorenzo Travaglini[a], Stefano Lai[b], Luca Patruno[c], Stefano de Miranda[c], Annalisa Bonfiglio[b], Piero Cosseddu[b], Beatrice Fraboni[a]

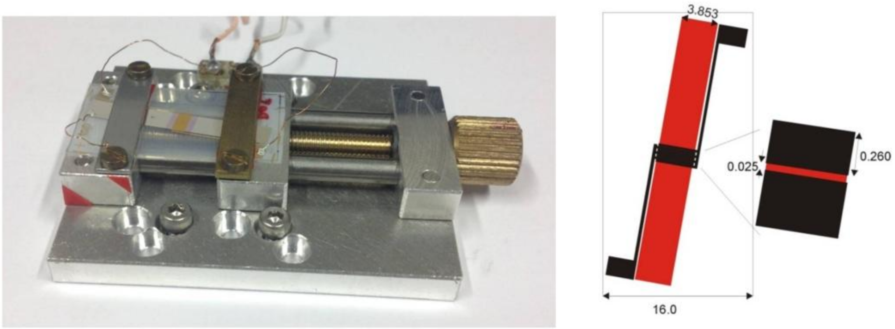

Figure S1: AFM sample holder and transistor layout: (a) optical migrograph of sample holder and mounted organic transistor. (b) geometry of gate (red) and source and drain electrodes (black).

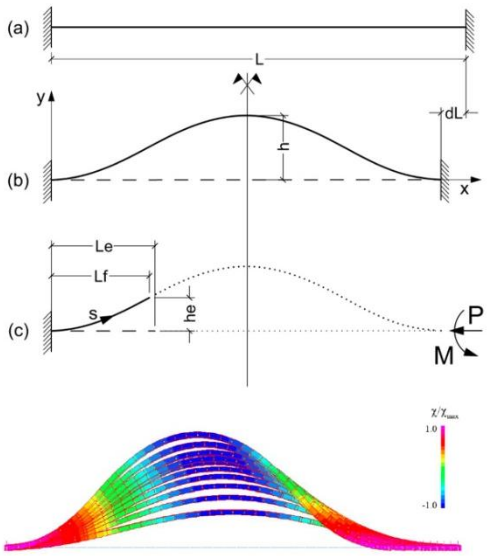

Figure S2: Model for OTFT deformation: (a-c) Geometric parameters used in the mathematical description of transistor deformation. (d) Bent substrate and color coded the resulting surface strain distribution.

Mathematical Model for substrate deflection

The deformation of homogeneous elastic beams considering large deflections has been deeply investigated in the literature, see for example [1]. In this context, the OTFT under examination can be studied by considering a cantilever beam with length $L_e = L/4$ loaded with a horizontal force P, as shown in Fig. S2 (the complete clamped system is then obtained by considering four cantilever beams aligned). In this case, it is possible to write [1]:

$$L_e = \frac{1}{k} K(p), \tag{S1}$$

where $k^2 = P/EI$, being E the Young's modulus and I the bar cross-section inertia, p is a parameter such that $p \in [0,1]$ and $K(p)$ is the complete elliptic integral of first kind defined as:

$$K(p) = \int_0^{\frac{\pi}{2}} \frac{\mathrm{d}\varphi}{(1 - p^2 \sin^2 \varphi)^{\frac{1}{2}}}. \tag{S2}$$

Assuming k and p as known parameters, it is possible to calculate the maximum deflection of the cantilever beam, h_e, the position of the tip along the x-axis, L_f, and its maximum curvature, $\chi_{max} = 1/r_{c\,min}$, as:

$$h_e = \frac{2p}{k}\ (a), \quad L_f = \frac{2E(p)}{k} - L_e\ (b), \quad \chi_{max} = -\frac{2pK(p)}{L_e}\ (c), \tag{S3}$$

being $E(p)$ the complete elliptic integral of second kind defined as:

$$E(p) = \int_0^{\pi/2} (1 - p^2 \sin^2 \varphi)^{1/2} \mathrm{d}\varphi. \tag{S4}$$

In order to obtain p, when the force P and the mechanical properties of the bar are known, it is possible to invert Eq. (S1) numerically. The value of p obtained in such a way can be then substituted in Eq. (S3) in order to obtain the main quantities of interest. When a system composed of a beam clamped at both ends is considered and a displacement is imposed at one of the clamps, it is necessary to proceed as follows. Firstly, Eq. (S3b) is rewritten as:

$$2L_e - dL_e = \frac{2E(p)}{k}, \tag{S5}$$

where $dL_e = L_e - L_f$ is the horizontal displacement applied at the cantilever beam tip. Then, k is obtained from Eq. (S1) and substituted in Eq. (S5) obtaining:

$$\frac{E(p)}{K(p)} = 1 - \frac{dL_e}{2L_e}. \tag{S6}$$

Such equation can be inverted numerically providing the value of p and, by means of Eq. (S3), the parameters of interest. The geometrical relations needed to transform the results obtained for the cantilever beam into the quantities for the clamped system are:

$$h = 2h_e, \quad dL = 4\, dL_e. \tag{S7}$$

Results obtained by means of a geometrically nonlinear Finite Element Analysis (FEM) which reproduce the mechanical behavior of the clamping device are reported in Fig. S2. As expected, comparison between the reported analytical solution and the FEM results showed an almost perfect agreement and so it is not here reported for brevity.

In order to simplify the strain determination in bend devices, a simplified relation between the clamp displacement and the maximum curvature is here proposed based on a best fit of the analytical solution:

$$\chi_{max} L = 0.81 + 13.23\frac{dL}{L} + 2.5\left(1 - e^{-15.35\frac{dL}{L}}\right). \tag{S8}$$

Notice that such approximated relation is accurate within approximately 3% error in the range $0.01 < dL/L < 0.9$.

[1] Frisch-Fay, R. (1962). Flexible bars. Butterworths, London.
